# The complete mitochondrial genome sequence and annotation of *Tylopilus plumbeoviolaceoides* T.H. Li, B. Song & Y.H. Shen, 2002 (Boletaceae, Boletoideae)

**DOI:** 10.1080/23802359.2022.2079104

**Published:** 2022-06-12

**Authors:** Wenbo Shi, Weicai Song, Yuan Peng, Shuo Wang, Guiwen Yang, Chao Shi

**Affiliations:** a College of Marine Science and Biological Engineering, Qingdao University of Science and Technology, Qingdao, China; b Shandong Provincial Key Laboratory of Animal Resistance Biology, College of Life Sciences, Shandong Normal University, Jinan, Shandong, China; c Plant Germplasm and Genomics Center, Germplasm Bank of Wild Species in Southwest China, Kunming Institute of Botany, The Chinese Academy of Sciences, Kunming, China

**Keywords:** *Tylopilus plumbeoviolaceoides*, Boletaceae, mitochondrial genome, phylogenetic relationships

## Abstract

*Tylopilus plumbeoviolaceoides* T.H. Li, B. Song & Y.H. Shen, 2002 is a species of basidiomycete in the family Boletaceae and is mainly found in Yunnan and Guangdong provinces in China. In this study, the mitochondrial genome of *T. plumbeoviolaceoides* was reported for the first time. The total length of the mitochondrial genome of *T. plumbeoviolaceoides* was 37,242 bp, with GC content of 23.0%. The mitochondrial genome of *T. plumbeoviolaceoides* contained 14 conserved protein-coding genes, 25 transfer RNA genes, and 2 ribosomal RNA genes. The phylogenetic tree indicated that *T. plumbeoviolaceoides* was closely related to *Xerocomus impolitus* and *Butyriboletus roseoflavus.* The complete mitochondrial genome of *T. plumbeoviolaceoides* will be useful for future research on basidiomycetes.

*Tylopilus plumbeoviolaceoides* T.H. Li, B. Song & Y.H. Shen, 2002 is a species of basidiomycete in the family Boletaceae and is mainly found in Yunnan and Guangdong provinces in China. A purple cap and a white stalk are distinguishing features of *T. plumbeoviolaceoides* (Falandysz et al. 2016; Rodríguez-Ramírez et al. 2020). *Tylopilus plumbeoviolaceoides* has a bitter taste and may cause diarrhea (Gelardi et al. 2015). The mitochondrial genome has a wide range of applications in the study of fungal species phylogeny (Zhang et al. 2017). However, the mitochondrial genome of *T. plumbeoviolaceoides* has not yet been reported. In this study, the mitochondrial genome of *T. plumbeoviolaceoides* was reported for the first time. We analyzed the general features of the mitochondrial genome of *T. plumbeoviolaceoides* and performed a phylogenetic analysis.

The specimen of *T. plumbeoviolaceoides* was sampled from Panlong District, Kunming City, Yunnan Province, China (24°23'N, 102°10'E). This research was conducted with the permission of the local government and the Kunming Institute of Botany, Chinese Academy of Sciences. The voucher samples and genomic DNA were stored at Qingdao University of Science and Technology (Chao Shi, chsh1111@aliyun.com) under the specimen code TP0202109. The 500 bp genome sequencing libraries were constructed for *de novo* sequencing. These libraries were sequenced on the Illumina HiSeq platform (Illumina, San Diego, CA, USA) in Novogene (Beijing, China). The complete mitochondrial genome of *T. plumbeoviolaceoides* was assembled using NOVOPlasty v4.3.1 (Dierckxsens et al. 2017) and was annotated by MFannot that was used in previous studies (Zhang et al. 2017). The software of tRNAscanSE v1.21 (Schattner et al. 2005) was used to detect tRNA genes under the default settings, and RNAmmer (Lagesen et al. 2007) was used to validate rRNA genes under the default settings. Sequin was used to manually correct codons and gene boundaries.

The complete mitochondrial genome of *T. plumbeoviolaceoides* was a typical circular molecule of 37,242 bp in length, with GC content of 23.0% (GenBank accession MW660363). The mitochondrial genome of *T. plumbeoviolaceoides* contained 20 putative protein-coding genes, including 14 conserved protein-coding genes (PCGs) and 6 open reading frames (ORFs) of unknown function. There were 25 transfer RNA (tRNA) genes in the mitochondrial genome of *T. plumbeoviolaceoides*, with 20 of which were unique. The 25 tRNA genes used 22 codons covered all 20 standard amino acids. There were two ribosomal RNA (rRNA) genes, small ribosomal RNA (*rns*) and large ribosomal RNA (*rnl*). The mitochondrial genome had a base composition of A (39.4%), C (11.4%), G (11.6%), and T (37.6%). The 14 conserved proteins were comprised of 7 NAD subunits dehydrogenases (*nad1*–*6* and *nad4L*), 3 cytochrome oxidases (*cox1*–*3*), apocyto chrome b (*cob*), and 3 ATP synthases (*atp6*, *apt8*, and *apt9*). We constructed A phylogenetic tree based on mitochondrial protein-coding genes of 12 Boletales species from the NCBI database to reveal the phylogenetic relationships between *T. plumbeoviolaceoides* and other Boletales species. *Moniliophthora perniciosa* and *Moniliophthora roreri* were used as outgroup. To create sequence alignments for the construction of phylogenetic trees, MAFFT v725 (Katoh and Standley 2013) was applied to protein-coding genes. Then, the GTR-GAMMA (GTR + G) model was identified as the best fitting substitution model by applying the Bayesian Information Criterion (BIC) using Modeltest (Posada and Crandall 1998). Lastly, phylogenetic trees, with branch support based on 1000 bootstrap replicates, were inferred using the maximum likelihood (ML) method as implemented in MEGA-X software (Kumar et al. 2018). The phylogenetic tree indicated that *T. plumbeoviolaceoides* was closely related to *Xerocomus impolitus* and *Butyriboletus roseoflavus* than to other Boletales species (Figure 1). The mitochondrial genome sequence of *T. plumbeoviolaceoides* will contribute to future research on basidiomycetes.

**Figure 1.**
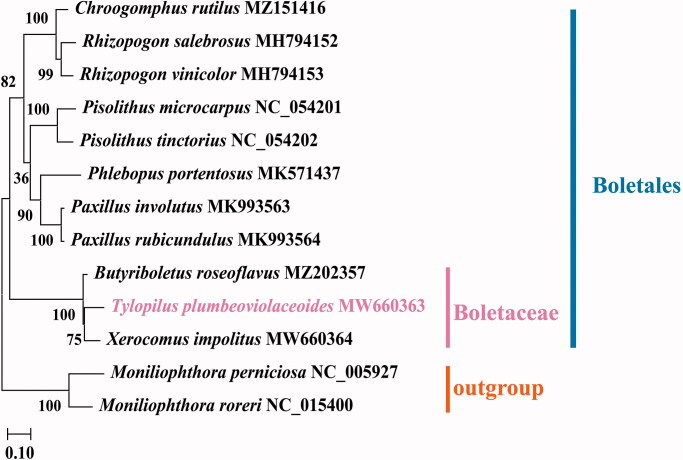
The maximum likelihood tree states the phylogenetic position of *T. plumbeoviolaceoides* in family Boletaceae, with the number on each node denoting the bootstrap support value. The species is followed by the chloroplast genome sequence accession number that was used by GenBank.

## Data Availability

The genome sequence data that support the findings of this study are openly available in GenBank of NCBI at (https://www.ncbi.nlm.nih.gov/) under the accession MW660363. The associated BioProject, SRA, and Bio-Sample numbers are PRJNA786973, SRR17162356, and SAMN23765445, respectively.
